# Gibberellic acid modulates drought stress signaling, antioxidant defense, and ionic homeostasis in spinach

**DOI:** 10.1080/15592324.2026.2664969

**Published:** 2026-05-09

**Authors:** Sadia Ashraf, Muhammad Shahbaz, Tahrim Ramzan, Arslan Haider, Muhammad Ahmad, Hossam S. El-Beltagi, Irsa Rahim, Mahrukh Nasir, Usman Zulfiqar, Gamal Awad El-Shaboury, Abdulrahman Alasmari, Elsevar Asadov, Mohd Asif Shah

**Affiliations:** a Department of Botany, University of Agriculture, Faisalabad, Pakistan; b West Central Research Extension and Education Center, University of Nebraska-Lincoln, North Platte, NE, United States of America; c Department of Agronomy & Horticulture, University of Nebraska-Lincoln, Lincoln, NE, United States of America; d Agricultural Biotechnology Department, College of Agriculture and Food Sciences, King Faisal University, Al-Ahsa, Saudi Arabia; e Department of Agronomy, Faculty of Agriculture and Environment, The Islamia University of Bahawalpur, Bahawalpur, Pakistan; f Department of Biology, Nakhchivan State University, Nakhchivan, Azerbaijan; g Biology Department, College of Science, King Khalid University, Abha, United States of America; h Department of Biology, Faculty of Science, University of Tabuk, Tabuk, Saudi Arabia; i Biodiversity Genomics Unit, Faculty of Science, University of Tabuk, Tabuk, Saudi Arabia; j Department of Basic Medical Sciences, Nakhchivan State University, Nakhchivan, Azerbaijan; k Kardan University, Kabul, Afghanistan; l Division of Research and Development, Lovely Professional University, Phagwara, Punjab, India; m University Centre for Research & Development, Chandigarh University, Gharuan, Mohali, Punjab, India

**Keywords:** Antioxidants, gibberellic acid, ionic contents, osmolytes, spinach

## Abstract

Drought stress is a major environmental signal that disrupts plant growth and metabolic homeostasis, particularly in water-sensitive leafy vegetables, such as spinach (*Spinacia oleracea* L.). Plant hormones play a central role in decoding stress signals and coordinating adaptive responses. This study investigated the role of exogenous gibberellic acid (GA) in regulating drought-induced physiological, biochemical, and ionic signaling in spinach. A pot experiment was conducted using two spinach cultivars (V1 = Desi, V2 = Lahori) subjected to well-watered and drought conditions (50% field capacity), combined with foliar GA applications (0, 100, and 200 ppm). Drought stress markedly altered plant behavior by suppressing growth traits, photosynthetic pigment synthesis, and redox balance, while increasing reactive oxygen species accumulation. GA application, particularly at 200 ppm, significantly modulated drought stress signaling by activating antioxidant defense systems [CAT (51.1%), POD (44.2%), and SOD (42.6%)], reducing oxidative damage indicators [H₂O₂ (3.6%) and MDA (25.3%)], and restoring metabolic stability. In addition, GA regulated ionic homeostasis by limiting Na⁺ (11.2%) accumulation and promoting K⁺ (96.6%) and Ca²⁺ (46.3%) uptake in both roots and shoots, reflecting improved stress adaptation. Cultivar-specific responses indicated higher signaling sensitivity and adaptive capacity in the Desi variety under drought stress. Overall, these findings demonstrate that gibberellic acid acts as a key regulatory signal that orchestrates antioxidant, metabolic, and ionic responses, thereby increasing drought stress adaptability in spinach.

## 1. Introduction

Climate change has significantly reduced the amount of agricultural output worldwide. As a result of climate change, crops are more vulnerable to abiotic stresses, including drought, salinity, temperature stress (heat stress, chilling, and freezing), waterlogging, and heavy metal accumulation.^1^ The wide range of stresses that cause an annual 50% reduction in agricultural productivity, it is known as drought stress.^2^^,^^3^ Drought stress in crop fields is a common occurrence because of the highly uneven distribution of effective rainfall during the season in rainfed areas. Drought stress is common in crop fields.^4^^,^^5^ A major influence on the sustainability of global food production is drought, which is mediated by water restrictions.^6^^,^^7^ Low nutrient levels, inadequate photosynthesis, and scarce water resources all contribute to reduced crop growth and productivity under conditions of water stress.^8^^,^^9^ The drought stress reduces photosynthesis by degradation of chlorophyll, stomatal closure, and inhibits the generation and buildup of energy sources by interfering with the electron transport and making it harder for the plant to absorb light.^10^

Drought stress reduction in agricultural productivity is mostly caused by a change in biochemical and physiological processes, demonstrating that stressed plants produce and store more ethylene and abscisic acid (ABA), which reduces plant productivity.^9^^,^^11^^,^^12^ Plants that are under water stress produce large amounts of reactive oxygen species, which set off oxidative damage mechanisms, such as lipid membrane peroxidation.^13^^,^^14^ Changes in the quantity and size of leaves, water relations, root growth, and stem elongation are all indicators of an impending drought^15^ through preventing photosynthetic arrest, disrupting the development of tissue hydration, aberrant metabolism, stomatal closure, and reducing cell division, all of which led to the termination of the cell's growth or death.^16^

Spinach (*Spinacia oleracea* L.) is a dioecious plant, with a maximum height of 30 cm, belongs to the Chenopodiaceae family.^17^ This leafy green crop has high economic value and is frequently grown. The first records of spinach cultivation date back to ancient Persia, about 2000–3000 y ago.^18^ The amount of spinach produced worldwide each year is 32 million tons.^19^ Generally, spinach is an annual plant that occasionally becomes biennial.^20^ It's primary constituents are protein (2.9%), fat (0.4%), water (91.4%), and carbs (3.6%). In addition to having higher amounts of iron, potassium, and magnesium, a 100-g serving of spinach meets 15%, 16%, and 20% of the daily recommended need.^21^ In addition to its commercial value, spinach contains high folate and vitamin contents, including K, C, and A.^22^^,^^23^ A major drop in the productivity of spinach plants was detected in water-scarce conditions; in particular, 50% irrigation showed a notable suppression of crop output.^24^ To maximize spinach yield while preserving its high nutritional value, creative methods are required. A possible solution for this problem is the targeted use of plant growth regulators, which are well-known for their regulatory functions in the physiological processes, development, and growth of plants.^25^^,^^26^

Many studies have shown that when plants are treated with growth-promoting hormones, such as auxin, gibberellins, brassinosteroids, and cytokinins, they become less resistant to water shortage.^27^^,^^28^ Among these are growth hormones called gibberellins (GAs), which are without a doubt engaged in a variety of physiological activities.^29^ Activating secondary metabolism during drought stress and possibly controlling water balance are two functions of plant growth regulators. Gibberellic acid, or gibberellin, is primarily used to stimulate plant growth, but it also serves as a stress-resistance agent, including water scarcity.^30^^,^^31^

Gibberellic acid stimulates cell growth, leaf and stem elongation, photosynthetic capability, flower production, uniform flowering, and enhances the size and rise in number of flowers.^32^ Gibberellic acid can encourage fruit senescence, blossoming, root elongation, flowering, and leaf expansion in addition to seed germination or dormancy.^33^ Gibberellin influences how plants store and utilize starch, which may have an effect on the plant's overall growth and reduces the water potential of the cell, permitting it to take more water and enhancing its resilience to water stress.^32^ It has been found that exogenously applying GA accelerates flowering, lengthens and multiplies siliqua and inflorescence, enhances plant height and leaf arrangement, and increases stem diameter and extension.^34^ GA accumulation is prevented by osmotic stress, which also regulates many other aspects of plant growth, including stem elongation, germination, flowering time, fruit development, and overall plant growth.^27^

Although water deficit causes oxidative damage by generating reactive oxygen species, GA is known to regulate several physiological processes. However, its role in enhancing drought stress tolerance through antioxidant activation and ionic balance in leafy vegetables, such as spinach, has not been comprehensively elucidated. Therefore, a detailed physiological and biochemical investigation is needed to understand how foliar-applied GA can modulate multiple defense components to improve drought resilience in spinach. This study hypothesized that exogenously applied GA could enhance the antioxidant defense system and promote metabolite accumulation, thereby improving the morphological and physiological attributes of spinach. The goal is to determine how exogenous GA contributes to drought-tolerant mechanisms in spinach through morphological, physiological, biochemical, and ionic adjustments.

## 2. Materials and methods

A pot experiment was conducted at the Old Botanical Garden of the University of Agriculture, Faisalabad, Pakistan. The experiment was arranged during the years 2023–2024 under a CRD (completely randomized design) with three replications. Treatment of the study was V1 = Desi, V2 = Lahori, drought stress (control and 50% FC), and GA (0, 100, and 200 ppm). The spinach seeds of two varieties were picked up from the Ayub Agricultural Research Institute (AARI), which is situated in Faisalabad, Pakistan. For this, 36 pots were used, with half of the pots containing Desi seeds and the other half containing Lahori seeds. Each variety was sown in plastic pots with dimensions (28 cm × 24 cm × 21 cm) containing 8 kg of soil in each pot and the soil pH was 7.7, and the EC was 4 dS m^−1^. In each pot, 18 seeds were planted. Six seedlings of the same size were kept in each pot by thinning after a week of germination. Irrigation was usually applied every 2–3 d. After 45 d of sowing, when the seedlings became established, two drought stress levels (control and 50% FC) were applied to the plants. After the maintenance of drought stress conditions for 2 weeks, three levels of GA (0, 100, and 200 ppm) as a foliar spray were applied after 60 d of sowing to determine which was best to ameliorate the effect of water shortage in spinach plants. Foliar application of GA was applied after sunset to minimize the effect of evapotranspiration. Control plants were sprayed with distilled water. Harvesting was done after 3 months of sowing.

### 2.1. Determination of field capacity

One hundred grams of soil sample was taken at pot filling time and dried in an envelope by placing it in the oven at 70 °C for 14 d (up to constant weight). Once the soil had dried, its saturation process was measured by weight, and the field capacity was determined by the following formula:

Moisture contents(M.C)=Fresh weight−oven−dried weight,

M.C=100goffreshsoil−89.81gofdriedsoil,

$$\mathrm{FC}=\mathrm{Saturation}\;\mathrm{percentage}/2^{{}^{\circ}}.$$

### 2.2. Harvesting and data collection technique

After 3 months of seed sowing, harvesting of plants was accomplished. Two plants, along with their roots, were taken out of each pot for the morphological parameters. Samples of fresh plants were sealed tightly in plastic zip bags and stored in a freezer set at −20 °C to measure all physiological variables. For dry parameters, two samples with shoots and roots were taken from each pot, and these samples were stored in the oven at 65 °C for 15 d to be subjected to dry weight and ion analysis.

### 2.3. Determination of morphological parameters

The root and shoot fresh weight were determined instantly after the plant samples were harvested, by using a digital weighing balance (Model: OHAUS Corporation, USA). To find the root dry weight, the plants were properly labeled, air-dried, and then dried in an oven for 2 weeks. After the roots were dried, their dry weight was determined using the electronic weight balance (Model; OHAUS Corporation, USA).

### 2.4. Photosynthetic pigments

The Arnon^35^ approach was used to estimate the photosynthetic attributes. Fresh leaf material of 0.1 g was cut into tiny pieces for this experiment. Then, each sample was put into a sample container or small bottles with 5 mL of an 80% acetone solution. After that, keep these sample bottles overnight at room temperature (25 °C) to observe how the solution's color changes. A spectrophotometer (Model WE 721 WELab instrument limited) was then used to measure the wavelength of absorption of these solution samples at 663, 645, and 480 nm. The chlorophyll content was quantitatively calculated using the following formulas:

$$\mathrm{Chl}.\;a\;(\mathrm{mg}g^{-1}\mathrm{FW})\;=[12.7(\mathrm{OD}663)-2.69(\mathrm{OD}645)]\times W\times V/1000,$$

$$\mathrm{Chl}.\;b(\mathrm{mg}g^{-1}\mathrm{FW})=[(22.9(0D645)-4.68(\mathrm{OD}663)]\times W\times V/1000,$$

$$\mathrm{Carotenoids}(\mathrm{mg}g^{-1}\mathrm{FW})=[(\mathrm{OD}480)+0.114(\mathrm{OD}663)-0.638(\mathrm{OD}645)]/2500.$$

### 2.5. Reactive oxygen species determinants

A freshly harvested 0.25 g sample of vegetative matter from a plant was crushed in 3 mL of 0.5% TCA (trichloroacetic acid) (0.5 g TCA diluted in 100 mL of distilled water). Then, this solution was taken in an Eppendorf. Then, centrifuged the solution at 12,000 rpm for 12 min. After that, a different Eppendorf tube was used to separate the supernatant, kept in the freezer at −12 °C.

#### 2.5.1. Estimation of hydrogen peroxide (H_2_O_2_) contents

The hydrogen peroxide concentration was measured by using the procedure proposed by Velikova et al. ^36^ Test tubes were loaded with 0.5 mL of sample extract, 0.5 mL of potassium phosphate buffer prepared by adding 8.7 g of K_2_HPO_4_ and 6.8 g of KH_2_PO_4_ in 1 L of distilled water along with 1 mL of potassium iodide solution, which is made by dissolving 16.6 g of KI dissolved in 100 mL of distilled water. After vortexing this solution, measurements at 390 nm were obtained by using a spectrophotometer (Model: WE 721 WELab instrument limited).

#### 2.5.2. Determination of malondialdehyde (MDA) contents

The MDA concentration was determined by using the Heath and Packer^37^ technique. In a test tube, 1 mL of the obtained solution/extract was taken and followed by adding 1 mL of a 0.5% TBA solution in a 20% TCA solution (20 g TCA in 100 mL with 0.5 g TBA). After that, this solution was subjected to a temperature of 95 °C in a water bath for 15 min. Then, this solution was kept in the ice for 15 min. The readings were noted on a spectrophotometer (Model: WE 721 WELab instrument limited) at 532 and 600 nm.

### 2.6. Determination of enzymatic antioxidants

A 0.25 g fresh leaf sample was crushed in a K_3_HPO_4_ buffer for the enzyme extract. Fresh leaves of samples were ground in a 5 mL solution of phosphate buffer by using a mortar and pestle that has been chilled to a low temperature/ice-cold. After that, the prepared mixture was put into an Eppendorf tube. Following the grinding procedure, the obtained samples were centrifuged at 12,000 rpm for 15 min. The supernatant was extracted and used to measure each antioxidant's level of activity.

#### 2.6.1. Superoxide dismutase (SOD)

To measure SOD activity, Spitz and Oberley's^38^ bioassay was applied.

#### 2.6.2. Peroxidase (POD)

Using the Chance and Maehly^39^ technique, the activity of POD was measured.

#### 2.6.3. Catalase (CAT)

The Chance and Maehly^39^ technique was applied to detect the activity of catalase (CAT).

### 2.7. Determination of nonenzymatic antioxidants

#### 2.7.1. Estimation of ascorbic acid contents in leaf

The method of Mukherjee and Choudhuri^40^ was used to calculate the ascorbic acid content of leaves.

#### 2.7.2. Estimation of anthocyanin contents

The technique of Strack and Wray^41^ was utilized to estimate anthocyanin contents.

#### 2.7.3. Determination of flavonoid contents

Kim et al.^42^ technique was used to determine flavonoid content.

### 2.8. Estimation of osmolytes

#### 2.8.1. Total phenolics

To determine total leaf phenolic content, the Julkenen-Titto^43^ methodology was used.

#### 2.8.2. Quantification of total soluble proteins (TSP)

The Bradford^44^ technique was used to calculate TSP. For this, 0.25 g of fresh leaf of spinach.

#### 2.8.3. Total soluble sugars (TSS)

Yoshida et al.^45^ method was applied to calculate the total amount of soluble sugars.

### 2.9. Ion analysis of shoots and roots (Na^+^, K^+^, and Ca^2+^)

Dry shoot samples of 0.1 g were placed in digestion flasks, which were then filled with 3 mL of sulfuric acid. Samples were kept in the dark overnight. The next day, the flasks were kept on a hot plate, and H_2_O_2_ solution was added to them until the solution became colorless. Dilution was achieved by adding distilled water to digestion flasks and then filtering the solution into plastic bottles using Whatman's filter paper. After this, distilled water was added to the plastic bottles until the volume was up to 50 mL. The readings of several root and shoot ions, such as Ca^2+^, K^+^, and Na^+^, were noted by using a Sherwood flame photometer 410.

### 2.10. Statistical analysis

The collected data were evaluated and assessed by using a complete randomized design (CRD) with three replications. Co-stat software was used to assess the significance of the data.^46^ Statistix 8.1 was used to perform a three-factorial analysis of variance, and Tukey's test was used to compare means at the *p ≤ *0.05 level of significance. R-studio (V 4.3.3) was utilized to generate a heatmap and correlation matrix. Principal component analysis (PCA) was carried out using OriginPro2024 software, and Microsoft Excel (version 2016) (Microsoft Corporation, Redmond, WA, USA) was used to construct the graphs.

## 3. Results

### 3.1. Morphological parameters

Morphological characteristics of spinach showed highly significant (*p ≤ *0.001) results among both varieties under drought stress and gibberellic acid application (Table 1). The plants that were exposed to drought stress (50% FC) exhibited increase in root length by 43.2% and 40.4%, root fresh weight by 55.4% and 81.4%, and root dry weight by 60% and 46.3% in V_1_ and V_2_ but a reduction in shoot length, shoot fresh weight, and shoot dry weight (28.5%, 30%, and 33.6%) in V_1_ and (23.7%, 29.1%, and 36.4%) in V_2_ respectively, than control. Foliarly, use of 100 ppm GA increased the shoot fresh weight (22.7% and 28%) and dry biomass of shoots (22.3% and 34.6%), the root fresh weight (23.9% and 23.8%) and dry weight (26.1% and 25.7%), the root length (24.5% and 16.5%), and the shoot length (29.1% and 18.6%) at V_1_ and V_2_, respectively. While exogenously used, 200 ppm of GA increased the above morphological parameters, such as the fresh biomass of shoots up to 53.5% and 46.3%, the dry biomass of shoots up to 43.4% and 60.4%, the root fresh weight up to 46.3% and 43.9%, the root dry weight (44.3% and 50.5%), root length (42.4% and 30%), and shoot length (54.3% and 34.9%) in V_1_ and V_2_, respectively (Tables 1 and 2).

**Table 1.** Morphological parameters of *S. oleracea* were obtained by applying GA foliarly under water stress.

Varieties	Drought stress	Gibberellic acid (GA)	Fresh weight of shoot (g)	Dry weight of shoot (g)	Fresh weight of root(g)	Dry weight of root (g)	Root length (cm)	Shoot length (cm)
Desi	**Control**	Control	**9.16 ± 0.21**	**1.78 ± 0.02**	**0.55 ± 0.02**	**0.18 ± 0.03**	**3.23 ± 0.09**	**22.43 ± 0.47**
		GA (100 ppm)	**10.84 ± 0.25**	**2.12 ± 0.04**	**0.67 ± 0.01**	**0.24 ± 0.01**	**4.17 ± 0.15**	**26.53 ± 0.62**
		GA (200 ppm)	**13.18 ± 0.02**	**2.52 ± 0.02**	**0.81 ± 0.01**	**0.31 ± 0.01**	**5.33 ± 0.15**	**30.23 ± 0.65**
	**50% FC**	Control	**6.41 ± 0.25**	**1.18 ± 0.02**	**0.85 ± 0.01**	**0.29 ± 0.01**	**4.63 ± 0.12**	**16.03 ± 0.58**
		GA (100 ppm)	**7.86 ± 0.24**	**1.45 ± 0.05**	**1.05 ± 0.03**	**0.37 ± 0.01**	**5.77 ± 0.09**	**20.7 ± 0.29**
		GA (200 ppm)	**9.84 ± 0.26**	**1.70 ± 0.08**	**1.24 ± 0.02**	**0.42 ± 0.03**	**6.6 ± 0.15**	**24.73 ± 0.41**
Lahori	**Control**	Control	**11.35 ± 0.43**	**2.01 ± 0.05**	**0.56 ± 0.02**	**0.23 ± 0.01**	**4.03 ± 0.18**	**27.93 ± 0.52**
		GA (100 ppm)	**13.18 ± 0.37**	**2.47 ± 0.04**	**0.78 ± 0.01**	**0.31 ± 0.01**	**4.83 ± 0.18**	**31.47 ± 1.32**
		GA (200 ppm)	**15.18 ± 0.38**	**2.86 ± 0.08**	**1.02 ± 0.02**	**0.38 ± 0.01**	**5.67 ± 0.09**	**34.26 ± 0.85**
	**50% FC**	Control	**8.05 ± 0.25**	**1.28 ± 0.04**	**1.01 ± 0.04**	**0.34 ± 0.01**	**5.67 ± 0.15**	**21.3 ± 0.06**
		GA (100 ppm)	**10.30 ± 0.16**	**1.72 ± 0.09**	**1.25 ± 0.02**	**0.42 ± 0.01**	**6.6 ± 0.15**	**25.27 ± 0.49**
		GA (200 ppm)	**11.77 ± 0.31**	**2.05 ± 0.05**	**1.45 ± 0.02**	**0.51 ± 0.01**	**7.37 ± 0.26**	**28.73 ± 0.69**

Values represented mean **± **standard error of three replicates of spinach varieties that share the different lettering for a parameter showed significant variation at a significance level of *p* ≤ 0.001. V1 = Desi, V2 = Lahori, control = no drought stress and drought stress = 50% FC, control = No GA, GA = Gibberellic acid (100 ppm), GA = Gibberellic acid (200 ppm).

**Table 2.** Response of *S. oleracea* were obtained by applying GA foliarly under water stress.

	Response	
Attributes	Desi	Lahori
Fresh & dry weight of shoot	Maximum increment under 200 ppm of GA	Maximum increment under 50% FC at 200 ppm of GA
Fresh & dry weight of root	Enhanced under drought stress at 50% FC	Maximum increment under 50% FC at 200 ppm of GA
Root length	Enhanced under 50% FC	Maximum increment under drought stress at 200 ppm of GA
Shoot length	Decreased under drought stress at 50% FC	Maximum increment under 200 ppm of GA
Total Chl. Carotenoids	Increased with 100, 200 ppm of GA	Maximum increment with 100, 200 ppm of GA
H_2_O_2,_ MDA	Increased with 50% FC	Maximum increment with 50% FC
CAT, POD, SOD	Maximum increment with 100, 200 ppm of GA	Increased with 50% FC
TSP, TSP, AsA, total phenolics	Increased with 50% FC	Maximum increment with 100, 200 ppm of GA
Anthocyanins	Maximum increment with 100, 200 ppm of GA	Maximum increment with 100, 200 ppm of GA
Flavonoids	Maximum increment with 100, 200 ppm of GA	Maximum increment with 100, 200 ppm of GA
Shoot and root Na^+^	Increased with 50% FC	Increased with 50% FC
Shoot and root Ca^2+^	Maximum increment with 100, 200 ppm of GA	Maximum increment with 100, 200 ppm of GA
Shoot and root K^+^	Maximum increment with 100, 200 ppm of GA	Maximum increment with 100, 200 ppm of GA

### 3.2. Photosynthetic pigments

**Figure 1.**
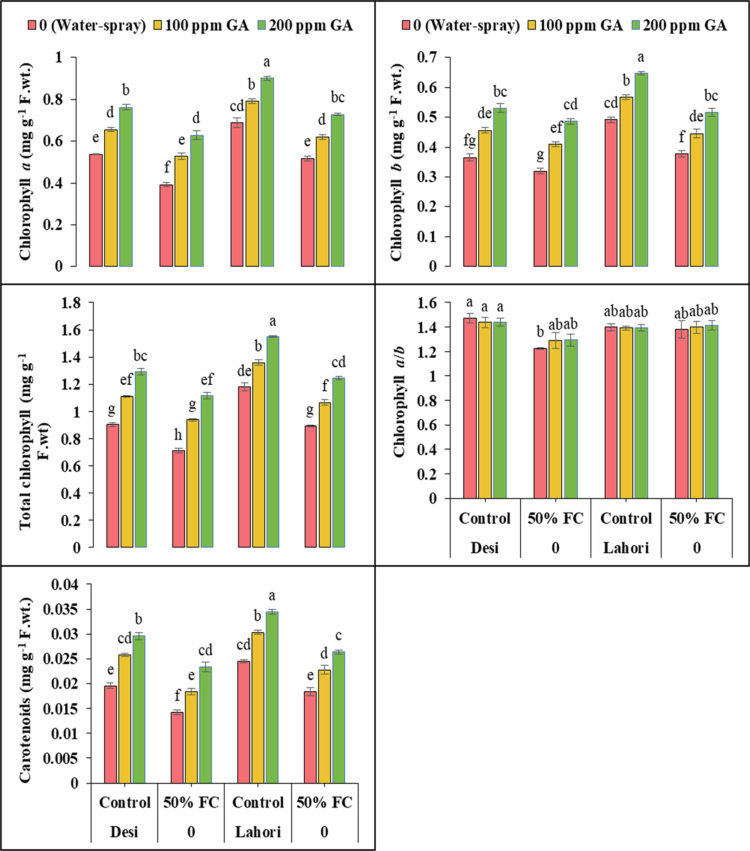
Gibberellic acid's effects on chlorophyll *a* (mg g^−1^ FW), chlorophyll *b* (mg g^−1^ FW), total chlorophyll (mg g^−1^ FW) under drought stress. At a high significance level of *p ≤ *0.001, bars that have identical letters for a parameter do not show any significant variations.

**Scheme 1.**
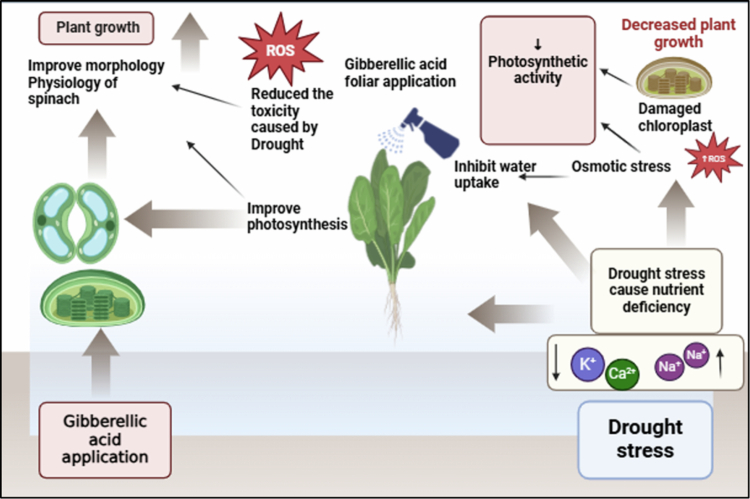
Foliar application of gibberellic acid modulates the morpho-physiological and ionic attributes of spinach plant under drought stress conditions.

Photosynthetic pigments except Chl. *a*/*b* displayed highly significant (*p ≤ *0.001) variations among both varieties of spinach under water deficit condition (50% FC) and GA application (Figure 1, Scheme 1). Chlorophyll *a*/*b* showed nonsignificant results. Under drought stress, photosynthetic pigments decreased significantly (*p ≤ *0.001). Photosynthetic pigments decreased up to chlorophyll *a* (27.1% and 24.7%), chlorophyll *b* (12.4% and 23.4%), total Chl. (21.1% and 24.2%), and carotenoids (27.5% and 24.9%) in V_1_ and V_2_, correspondingly under drought stress. Exogenously applied 100 ppm of GA increased the Chl. *a* (34.7% and 19.8%), Chl. *b* (28.3% and 18.1%), total chlorophyll (31.8% and 19.1%), and carotenoids (29.9% and 23.8%) in V_1_ and V_2_, respectively. However, the application of 200 ppm GA increased the Chl. *a* up to 60.5% and 40.5%, the Chl. *b* (52% and 36.9%), total chlorophyll (56.7% and 39%), and carotenoids (64.8% and 43.3%) in V_1_ and V2, respectively (Figure 1 and Table 2).

### 3.3. Reactive oxygen species determinants

**Figure 2.**
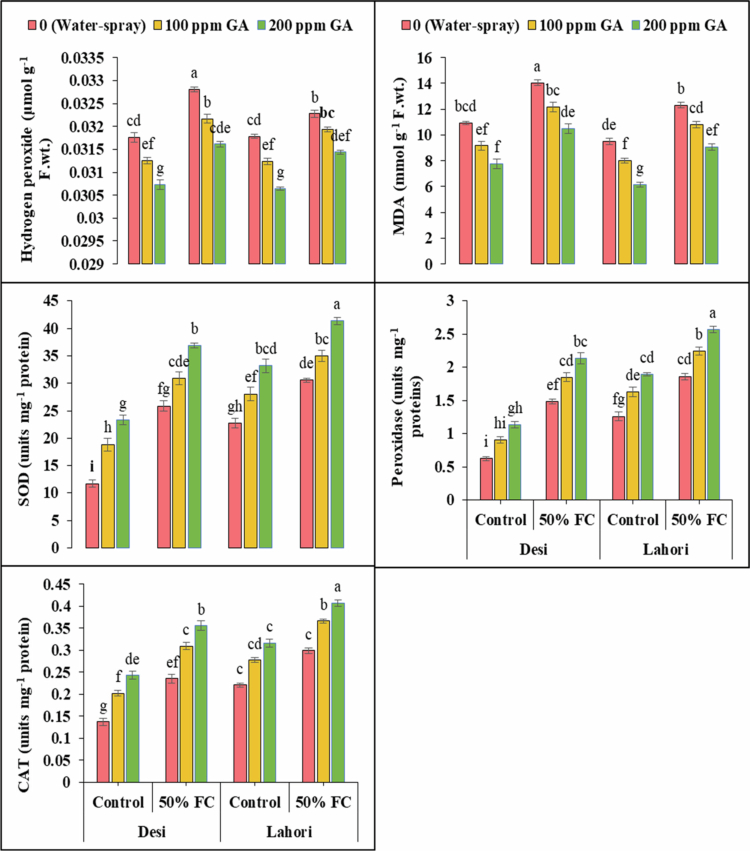
Gibberellic acid's effects on H_2_O_2_ (μmol g^−1^ FW), MDA (mmol g^−1^ FW), SOD (units mg^−1^ protein), POD (units mg^−1^ protein), and CAT (units mg^−1^ protein) under drought stress. At the significance level of *p *≤ 0.001, bars that have the identical letter for a variable do not show any prominent variations.

Hydrogen peroxide (H_2_O_2_) and malondialdehyde (MDA) exhibited highly significant (*p ≤ *0.001) results among both varieties (Desi and Lahori). These reactive oxygen species (ROS) of spinach showed highly significant (*p ≤ *0.001) results among both varieties under both drought stress and gibberellic acid application (Figure 2). The plants that were treated with water stress, enhanced contents of H_2_O_2_ and MDA. The H_2_O_2_ (3.3% and 1.6%) and MDA (28.4% and 29.7%) were increased in V_1_ and V_2_, respectively, under drought stress (50% FC). Foliarly used 100 ppm GA decreased H_2_O_2_ (2% and 1.03%) and MDA (13.3% and 12.2%) in V_1_ and V_2_ under drought stress (50% FC), respectively. Foliar application of 200 ppm GA decreased H_2_O_2_ (3.6% and 2.6%) and MDA (25.3% and 26.4%) in V_1_ and V_2_, respectively (Figure 2).

### 3.4. Enzymatic antioxidant

Peroxidase (POD), superoxide dismutase (SOD), and catalase (CAT) production were highly significant (*p *≤ 0.001) in both varieties of spinach (Figure 2). Under drought stress, these enzymatic antioxidants increased SOD (120.8% and 34.3%), POD (137.8% and 47.8%), and CAT (72.1% and 35.4%) in V_1_ and V_2_, respectively. By the use of 100 ppm GA, SOD (19.5% and 14.4%), POD (24.9 and 20.5%), and CAT (31.3% and 22.6%) increased in V_1_ and V_2_, respectively, in spinach. After applying the 200 ppm, GA showed high activity of SOD upto 42.6% and 35.3%, POD increased to 44.2% and 37.8%, and that of CAT increased to 51.1% and 36.1% at V_1_ and V_2_, respectively (Figure 2).

### 3.5. Nonenzymatic antioxidants

**Figure 3.**
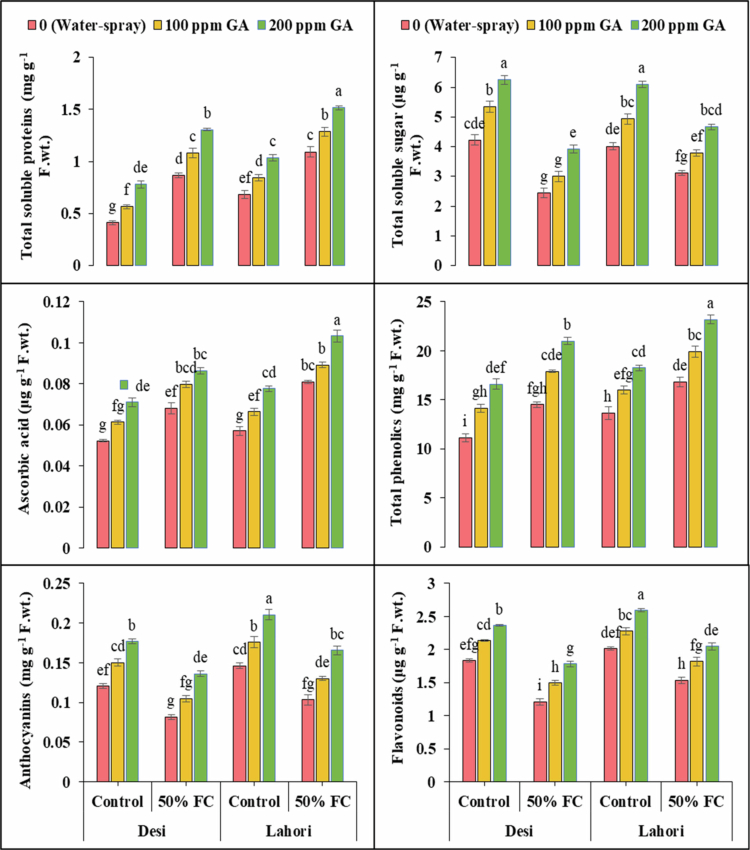
Gibberellic acid's effects on TSP (mg g^−1^ FW), TSS (μg g^−1^ FW), ascorbic acid (mg g^−1^ FW), total phenolics (mg g^−1^ FW), anthocyanins (mg g^−1^ FW), and flavonoids (μg g^−1^ FW) under drought stress. At the significance level of *p *≤ 0.001, bars that have the identical letter for variables that do not show any significant variations.

Ascorbic acid (AsA), phenolics, anthocyanins, and flavonoids showed highly significant variations (*p ≤ *0.001) in spinach (Figure 3). Ascorbic acid (30.7% and 41.7%) and phenolics (30.5% and 23.1%) increased in V_1_ and V_2_, respectively, under water deficit conditions (50% FC). (32.3% and 29.4%) and flavonoids (33.9% and 23.7%) decreased under water deficit conditions in V_1_ and V_2_, respectively. After the use of 100 ppm GA, ascorbic acid (17.1% and 10%), phenolics (23.2% and 18.6%), anthocyanin (28.2 and 25.8%), and flavonoids (23.4% and 18.3%) increased in spinach in V_1_ and V_2_, respectively. Gibberellic acid of 200 ppm increased the ascorbic acid (26.5% and 27.6%), phenolics (44.6% and 37.9%), anthocyanin (66.5% and 60.3%), and flavonoids (47.1% and 33.2%) in V_1_ and V_2_, respectively (Figure 3 and Table 2).

### 3.6. Osmolytes

Total soluble proteins (TSP) and total soluble sugars (TSS) contents showed highly significant (*p ≤ *0.001) variations in both varieties of spinach (Figure 3). Total soluble proteins content was observed more in V_1_ and V_2_ up to 109.5% and 60%, respectively, under drought stress. Exogenous application of GA (100 ppm) increased the TSP up to 25.1% and 17.6% at V_1_ and V_2_, respectively. Foliar application of 200 ppm GA increased the TSP up to 50.8% and 38.6% at V_1_ and V_2_, respectively. The TSS was decreased up to 42.2% and 22.6% at V_1_ and V_2_, respectively, under water stress conditions. After foliarly using 100 ppm GA increased TSS up to 23.1% and 22.2% at V_1_ and V_2_, respectively. Exogenous use of 200 ppm GA increased the total soluble sugars up to 60.6% and 50% in V_1_ and V_2_, respectively (Figure 3).

### 3.7. Inorganic ions

**Figure 4.**
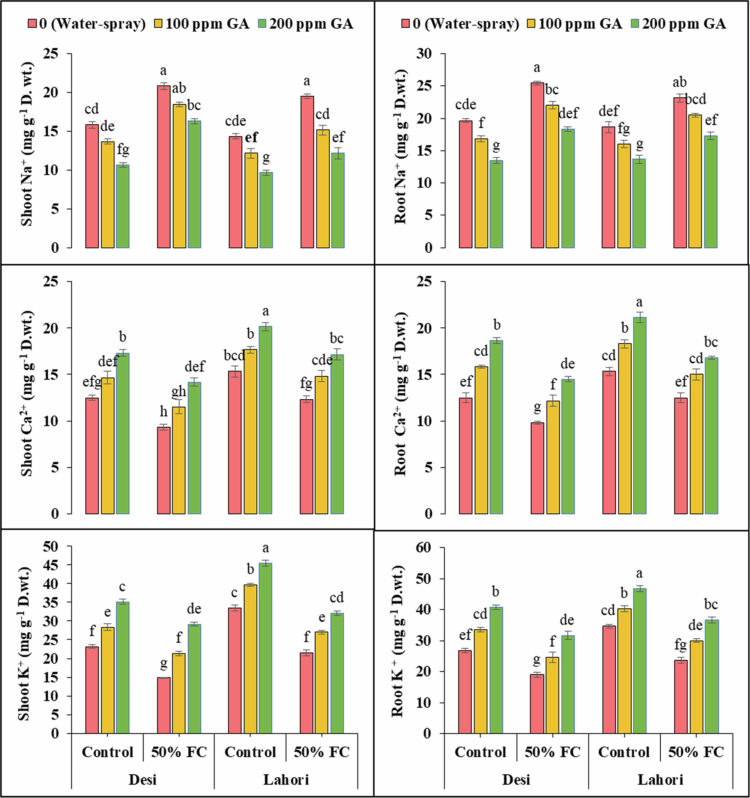
Gibberellic acid's effects on shoot Na^+^ (mg g^−1^ DW), root Na^+^ (mg g^−1^ DW), shoot Ca^2+^ (mg g^−1^ DW), root Ca^2+^ (mg g^−1^ DW), shoot K^+^ (mg g^−1^ DW), and root K^+^ (mg g^−1^ FW) under drought stress. At this significance level of *p *≤ **0.001, bars that have identical letters for a variable that does not show any prominent variations.

Shoot and root Na^+^ showed extremely significant (*p ≤ *0.001) behavior between the varieties of spinach (Figure 4). Shoot Na^+^ (31.6% and 36%) and root Na^+^ (29.7% and 24.1%) increased under drought stress (50% FC) in V_1_ and V_2_, respectively. The use of 100 ppm GA decreased the shoot Na^+^ (11.2% and 22.2%) and root Na^+^ (13.7% and 11.5%) in V_1_ and V_2_, respectively. After applying the 200 ppm GA, the shoot Na^+^ (21.6% and 37.6%) and root Na^+^ (28.1% and 25.2%) were reduced in V_1_ and V_2_, respectively. Shoot and root Ca^2+^ and shoot and root K^+^ showed slightly significant (*p *≤ 0.01) results between the spinach varieties (Figure 4). Shoot Ca^2+^ (25.3% and 19.6%), root Ca^2+^ (21.3% and 18.5%), shoot K^+^ (36% and 35.8%), and root K^+^ (29.2% and 31.7%) reduced significantly under water deficit conditions (50% FC) at V_1_ and V_2_, respectively. The use of 100 ppm GA increased shoot Ca^2+^ (23.2% and 20.3%), root Ca^2+^ (23.7% and 20%), shoot K^+^ (43.8% and 25.6%), and root K^+^ (29.8 and 26.8%) in V_1_ and V_2_, respectively. After exogenous application of 200 ppm GA increased shoot Ca^2+^ (51.8 and 39.2%), root Ca^2+^ (47.5% and 34.7%), shoot K^+^ (96.6% and 49.6%), and root K^+^ (66.7% and 54.9%) in V_1_ and V_2_, respectively (Figure 4 and Table 2).

### 3.8. Heatmap analysis

**Figure 5.**
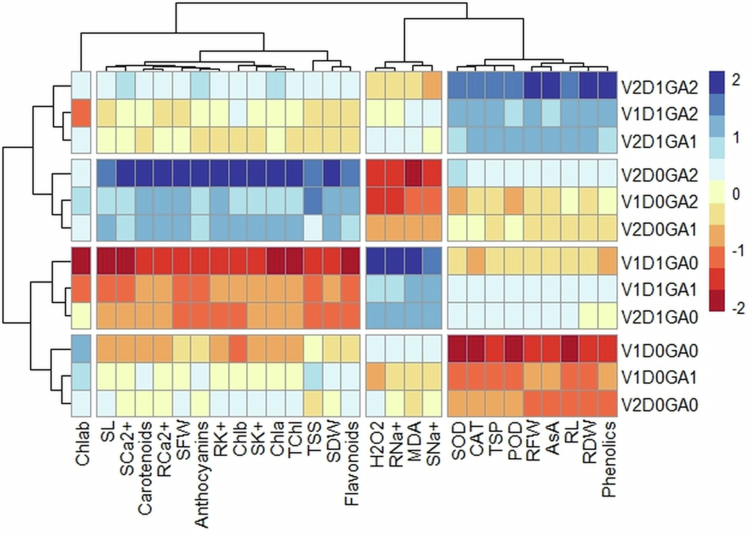
Heatmap with dendrogram showing positive and negative correlation of spinach varieties by application of GA under drought stress. V1 = Desi, V2 = Lahori, D0GA0 = control no drought + no treatment, D0GA1 = control + 100 ppm GA, D0GA2 = control + 200 ppm GA, D1GA0 = 50% FC + no treatment, D1GA1 = 50% FC + 100 ppm GA, D1GA2 = 50% FC + 200 ppm GA.

To detect the effects of GA on several parameters in spinach varieties under water deficit conditions, a two-way clustered heatmap was drawn (Figure 5). The link between the measurements was displayed by colored squares, and the measurements were grouped according to how similar they were at the different doses of treatments. Under drought stress conditions, the maroon color indicates a significant positive correlation, whereas the blue and light blue colors indicate a negative correlation of many parameters impacted by GA. Four categories have been identified using the heatmap. In the first group, phenolics, RDW, RL, SOD, POD, TSP, and CAT were clustered. A strong positive relationship between these parameters and drought in both varieties at application GA2 (200 ppm) and 50% FC, and negatively correlated under control conditions (no drought and treatment). Showing that the application of GA (200 ppm) helped in reducing drought stress by increasing enzymatic antioxidants. The second group containing the Shoot and root Na^+^, H_2_O_2_, and MDA. All these variables are strongly positive correlated with 50% FC and GA0 (no treatment) and negatively correlated and control (no drought) and GA2 (200 ppm), showing that GA helped in decreasing drought stress by decreasing Na^+^ ions, H_2_O_2_, and MDA content for mitigating harmful effects of oxidative damage. The third group containing flavonoids, SDW, total Chl., chlorophyll *b*, a, shoot and root K^+^ and Ca^2+^, SFW, carotenoids, shoot length and anthocyanins. These variables were highly positively correlated with the control (no drought) and GA2 (200 ppm), while showing a strong negative correlation with 50% FC and GA0 (no treatment) under drought stress. The fourth group containing Chl. *a*/*b* ratio, that is strong positively correlated at D0 and G0 (no drought and treatment). And negatively correlated at D1 (50% FC) and GA2 (200 ppm). These results exhibited that the use of GA (200 ppm) was beneficial for increasing growth features, ionic contents, photosynthetic attributes, enzymatic and nonenzymatic antioxidants, and osmolytes to induce tolerance against drought stress (Figure 5).

### 3.9. Correlation matrix and PCA analysis

**Figure 6.**
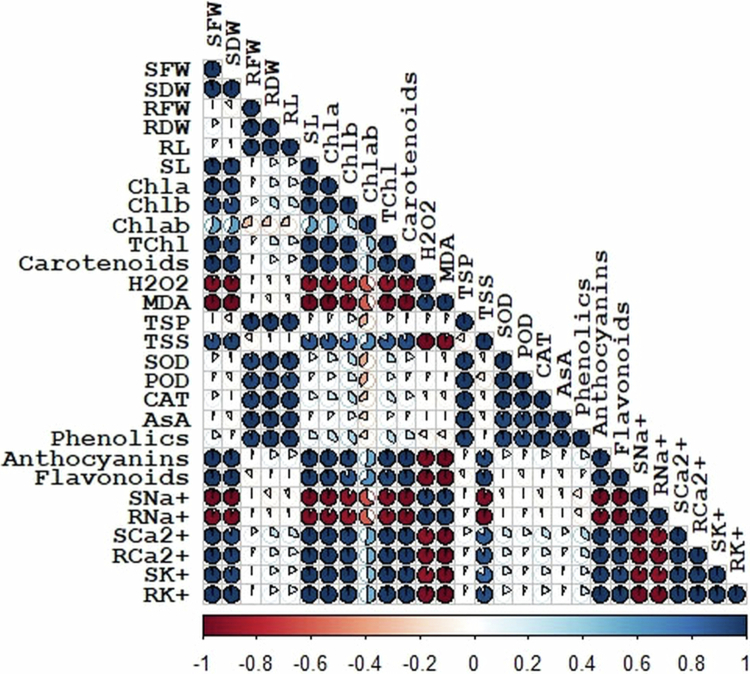
Correlation analysis among biochemical, morpho-physiological, and ionic features of spinach varieties under drought stress.

The matrix of correlation indicates strong negative and positive relationships between the measured spinach features under drought stress (Figure 6). The correlation revealed that growth parameters, such as shoot length, shoot fresh, and dry weight, are positively correlated with photosynthetic pigments, including chlorophyll and carotenoids, shoot and root K^+^, and shoot and root Ca^2+^. Furthermore, SOD, POD, CAT, phenolics, and AsA were all significantly negative. Additionally, H_2_O_2_, MDA, shoot, and root Na^+^ are all negatively correlated with growth and photosynthetic parameters (Figure 6).

**Figure 7.**
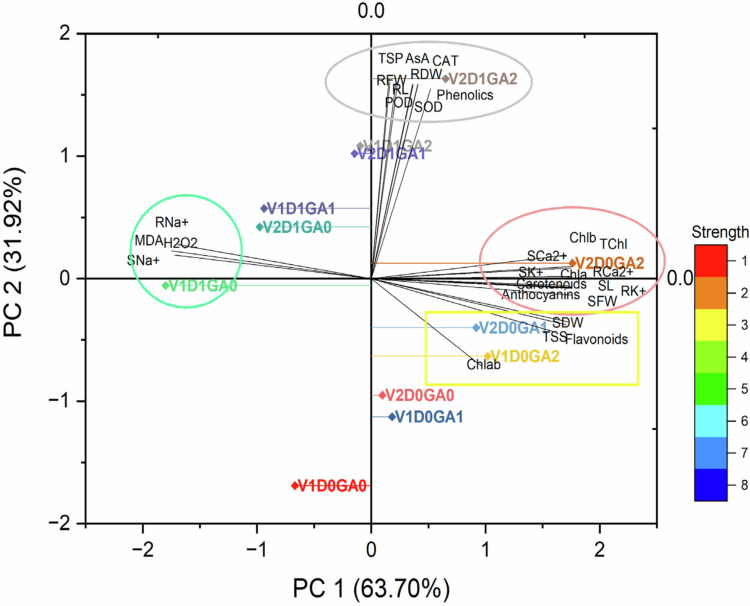
Principle component analysis of the morpho-physiological, biochemical, and ionic attributes of drought-stressed spinach varieties.

The PCA analysis revealed that PCA 1 and PCA 2 accounted for 95.62% of the accumulated variations, with 63.70% and 31.92%, respectively. However, the morphological, photosynthetic, enzymatic, and nonenzymatic antioxidants, as well as osmolytes, are positively clustered, whereas H_2_O_2_, MDA, shoot and root Na^+^ were greatly varied (Figure 7).

## 4. Discussion

Global climate change has created an intricate system of changing environmental situations, which is particularly critical because drought stress is increasing greater every day.^9^^,^^47^ Water stress is a significant problem affecting the yield of agricultural products that directly impacts food security.^48^ Compared to other environmental stresses, drought stress significantly decreases wheat growth and yield. Drought affects photosynthetic arrest, stomatal closure, the water potential of the tissue, reduced cell division, and abnormal metabolism, all of which lead to altered water relations, water use efficiency, leaf size, root growth, leaf number, and reduced stem expansion. These changes ultimately result in the suppression of growth.^49^^,^^50^ Gibberellins are plant hormones that are essential for plant development and growth because they promote pollen tube extension, cell division, pollen production, elongation, seed formation, germination, and fruit growth.^51^

According to the trial's results, spinach (*S. oleracea* L.) varieties Lahori and Desi showed a significant decrease in morphological parameters (such as shoot dry and fresh weights, shoot length, and root dry and fresh weights) under conditions of water scarcity (50% FC), while root length significantly increased under conditions of water stress (50% FC). The same findings were revealed in previous research on spinach,^52^ wheat,^53^ and pea^47^ that is because of lessened cell turgidity and decreased enzyme activity, which in turn led to decreased plant growth and cell division.^54^ Increasing the length of their roots helps plants better absorb water from the soil, which is one way they defend the plants against drought.^55^ Similar to our findings, GA significantly impacted the morphology and vegetative growth of coriander,^56^ wheat,^57^ and rice.^58^ This might be due to the fact that GA may have enhanced the amount of photosynthetic assimilates that were translocated to the vegetative organ, resulting in the enhancement of the growth of plants.^58^

In our findings, water stress prominently declined the photosynthetic pigments (Chl. *a*, *b*, total Chl., and carotenoids) in both spinach varieties (Desi and Lahori). Due to the reduction of carotenoid pigments brought on by drought stress, there is a drop-in chlorophyll. Drought also increase chlorophyll degrading enzyme called chlorophyllase by increasing ROS species which reduced the photosynthetic efficiency of crops. Consistent with our findings, in pea,^49^^,^^59^ spinach,^60^ and rice,^61^ chlorophyll contents were reduced only in the applications of water restrictions.^60^ The fall in chlorophyll content might be due to stomatal closure and structural deterioration of chlorophyll.^62^ The reduced chlorophyll content in plants may also be caused by the generation of oxygen-free radicals in unfavorable environments, which degrade pigments and ultimately reduce the quantity of chlorophyll in plants.^63^ The significant drop in chlorophyll content observed in pea leaves during periods of water scarcity may be attributed to the activation of enzymes that degrade chlorophyll, a malfunction in the photosynthetic apparatus, or both.^59^ The results of the current study were consistent with earlier research showing that cowpea plants treated with GA through foliar spray increased their levels of carotenoid and chlorophyll^63^ bread wheat^64^ and pea.^47^ It has been demonstrated that GA can prevent chlorophyll breakdown and eventually improve plant life.^63^

According to the results of a recent study, reactive oxygen species like MDA and H_2_O_2_ were markedly increased in drought stress environments by increasing electron leakage from chloroplast, ionic imbalance, and reducing membrane stability due to low availability of carbon dioxide. Similar results in pea plants were noted in earlier research,^65^^,^^66^ spinach,^60^ and maize^67^ as plants under drought stress experienced oxidative stress, which caused the accumulation of hydrogen peroxide and malondialdehyde. Reactive oxygen species (ROS) became high in stressed plants at the cellular level as a result of direct or indirect disturbances to metabolic processes.^67^^,^^68^ According to our research, exogenous GA application decreased MDA and H_2_O_2_ concentrations by increasing antioxidative activities and improving membrane stability. Previous data support our conclusion that applying GA spray to jute can yield similar benefits (*Corchorus capsularis* L.),^68^ spinach,^60^ and spring wheat^2^ decreased the amount of these compounds (MDA and H_2_O_2_), suggesting that it could be possible to mitigate drought with exogenous GA.^2^ Because it alters and decreases the activity of H_2_O_2_ metabolizing enzymes, it decreases lipid peroxidation and ion leakage of membranes, maintaining the plant and protecting the stability and integrity of the membrane under water stress.^63^

In the current trial, total soluble proteins increased in the presence of water deficit, while total soluble sugars decreased in spinach under water scarcity conditions. Soluble sugars are significantly impacted by environmental stresses because, under drought stress plant adapted a strategy of accumulation of solutes for maintaining a concentration gradient. Consistent with our results, another study suggested that water stress led to a notable decrease in rice's accumulation of soluble sugars^69^ and pea (*Pisum sativum* L.)^49^ because when photosynthesis is restricted by drought stress, starch is broken down to provide energy and carbon^69^, and TSP increased under water stress in *Pisum sativum* L.^47^ and spinach.^70^ Contradictory to our results, the synthesis of total soluble sugars increases in wheat^53^ to sustains the membrane's integrity, which prevents and delays membrane fusion and keeps proteins working. It is important for osmotic adjustment in an environment of water scarcity.^53^ Similar to our results, GA3 can increase the synthesis of osmoprotectants in wheat (*Triticum aestivum* L.),^71^ *Vicia faba,*^72^ and rice seedlings^73^ that help to maintain cell turgor and stability under water stress conditions.^71^ The total soluble protein was increased significantly under GA_3_ application,^74^ *Vicia faba.*^72^ Changes in protein synthesis, accumulation, and expression during water scarcity have been reported in earlier research; these changes are typically regulated by abscisic acid (ABA) hormones.^75^

According to current statistical data, the stress of drought considerably raised the levels of enzyme antioxidants, such as peroxidase (POD), superoxide dismutase (SOD), and catalase (CAT). Similar research revealed that water scarcity enhances the activity of SOD, CAT, and POD in *Brassica rapa,*^76^ and *Pisum sativum* L.^49^ by disturbing metabolic pathways.^67^ Drought stress and other environmental stressors cause an increase in CAT activity, which is vital for preventing oxidative damage and supporting SOD, APX, and other enzymes in reducing the harmful effects of reactive oxygen species. Similar findings from an earlier study demonstrated that the administration of GA3 dramatically increased the activities of CAT, POD, and SOD in jute (*Corchorus capsularis* L.),^68^ spring wheat,^2^ and *Vicia faba*^72^ showing that the Sesbania pea's^77^ capacity to tolerate abiotic stress was improved by the appropriate application of gibberellin, which in turn decreased the amount of extra active oxygen the plants produced and, to some extent, lessened or resisted the damage, allowing the plants to grow normally in the end.

The current study on spinach crop showed that water deficit conditions decreased the anthocyanins and flavonoids but nonenzymatic antioxidants, such as total phenolic and ascorbic acid (AsA) contents, increased. These findings are consistent with other studies, showing that phenolics increased during periods of water constraint in pea plants,^78^ spinach,^24^ and *Brassica napus,*^79^ under water stress AsA contents increased in spinach,^24^ wheat (*Triticum aestivum* L.),^71^ and *Vicia faba.*^72^ Ascorbic acid effectively reduces excessive formation of reactive oxygen species (ROS) and prevents oxidative stress^79^ and reduced anthocyanins under drought stress.^62^ Phenolics are hypothesized to be accumulated by plants within their tissues as a way to adapt the harsh environmental conditions. The function of chalcone synthase (CHS), phenylalanine ammonia lyase (PAL), and various other enzymes led to this accumulation. There are several physiological advantages that plant phenolics offer for withstanding and adapting to environmental stressors. Comparable to our findings, the application of GA increased AsA activity in broccoli (*Brassica oleraceae* L. var. *italica*)^80^ and increase the level of ascorbate in *Vicia faba*^72^ significantly increases the plant's resistance to oxidative stress.

Our study indicated that uptake of shoot and root Na^+^ ions highly increased under water scarcity condition (50% FC). While the shoot and root K^+^ and Ca^2+^ ions declined under water stress conditions. In our study, Na^+^ ions increased due to ionic imbalance caused by ROS species, and by reducing water in plant cells, Na^+^ ions became concentrated and cannot excluded from the roots. The same findings were found in past study in *Gossypium hirsutum* L.^81^, rice,^82^ and spinach.^70^ It is highly recognized in *Gossypium hirsutum* L.^81^ that increased Na^+^ concentrations can obstruct K^+^ uptake, which can lead to a reduction in plant dry matter and occasionally even death of plants. They investigated the relationship between the production of ROS and Na^+^ accumulation. Our findings that GA increased uptake of K^+^ and Ca^2+^ ions and decreased uptake of shoot and root Na^+^ ions in maize due to increased dilution of Na^+^ ions and it excluded from the roots. It correlate with previous studies.^74^ It has been noted that GA3 priming decreases Na^+^ buildup in plant tissues, which lessens the negative consequences of salt stress.^83^ Ion transporters, such as SOS1 (salt overly sensitive 1) and NHX (Na^+^/H^+^ antiporters), which aid in sodium exclusion from the cytoplasm and preserve ionic equilibrium within plant cells, are regulated to produce this effect. This implies that GA_3_ ability to either promote or hinder tiller formation depends critically on how it is applied.^84^^,^^85^

Our results indicate that GA applied topically strengthened both photosynthetic and morphological pigments. The GA applied topically increased the activity of total soluble sugars, flavonoids, anthocyanins, enzymatic and nonenzymatic antioxidants, and organic osmolytes under conditions of water scarcity. Furthermore, under the imposition of water shortage, the foliage application of GA markedly increased shoot K^+^ and Ca^2+^ while dramatically reducing shoot Na^+^.

## 5. Conclusion

Drought stress is one of the significant abiotic which harm the growth and development of spinach. While foliar application of gibberellic acid improved the morphological and photosynthetic parameters of spinach that were decreased under water scarcity stress conditions (Scheme 1). Drought stress increased the ROS levels, such as H_2_O_2_ and MDA, which had harmful consequences for plant growth and defense mechanisms. However, these characteristics were decreased by the use of foliar GA spray (0 ppm, 100 ppm, and 200 ppm). Foliar application of GA increased enzymatic and nonenzymatic antioxidants and the accumulation of osmolytes. The spinach plants' exposure to drought stress reduced their ability to uptake mineral ions, such as Ca^2+^ and K^+^, through the shoot and root, while increasing the level of Na^+^ ions. The foliar supplies of GA promoted the accumulation of mineral ions, such as Ca^2+^ and K^+,^ while decreasing the uptake of Na^+^ ions. The findings of current research indicate that foliar application of GA with 200 ppm was mitigated to reduce the negative effects of water stress on the Lahori variety of spinach as compared to the Desi variety. Gibberellic acid (GA) is inexpensive and has become readily available; hence, it has the potential of application in enhancing spinach growth during drought conditions. Although the pot experiment provides some controlled information, field tests are required to affirm the applicability of the findings in the actual agricultural environment.

## Data Availability

Data will be made available upon request.

## References

[1] Rai KK. Integrating speed breeding with artificial intelligence for developing climate-smart crops. Mol Biol Rep. 2022;49:11385–11402. doi: 10.1007/s11033-022-07769-4. 35941420 PMC9360691

[2] Al-Mahmud J, Biswas PK, Nahar K, Fujita M, Hasanuzzaman M. Exogenous application of gibberellic acid mitigates drought-induced damage in spring wheat. Acta Agrobot. 2019;72.

[3] Younis A, Ramzan F, Ramzan Y, Zulfiqar F, Ahsan M, Lim KB. Molecular markers improve abiotic stress tolerance in crops: a review. Plants. 2020;9:1374. 33076554 10.3390/plants9101374 PMC7602808

[4] Chen Y, Wang Y, Wu C, Jardim AMDRF, Fang M, Yao L, Liu G, Xu Q, Chen L, Tang X. Drought-induced stress on rainfed and irrigated agriculture: insights from multi-source satellite-derived ecological indicators. Agric Water Manage. 2025;307:109249. doi: 10.1016/j.agwat.2024.109249.

[5] Nadeem M, Li J, Yahya M, Sher A, Ma C, Wang X, Qiu L. Research progress and perspective on drought stress in legumes: a review. Int J Mol Sci. 2019;20:2541. doi: 10.3390/ijms20102541. 31126133 PMC6567229

[6] Ahmad W, Waraich EA, Haider A, Mahmood N, Ramzan T, Alamri S, Siddiqui MH, Akhtar MS. Silicon-mediated improvement in drought and salinity stress tolerance of black gram (*Vigna mungo* L.) by modulating growth, physiological, biochemical and root attributes. Am Chem Soc Omega. 2024;9:37231–37242. 10.1021/acsomega.4c04727 PMC11375724 39246467

[7] Khatun M, Sarkar S, Era FM, Islam AM, Anwar MP, Fahad S, Datta R, Islam AA. Drought stress in grain legumes: effects, tolerance mechanisms and management. Agronomy. 2021;11:2374. doi: 10.3390/agronomy11122374.

[8] Jabborova D, Annapurna K, Azimov A, Tyagi S, Pengani KR, Sharma P, Vikram KV, Poczai P, Nasif O, Ansari MJ, et al. Co-inoculation of biochar and arbuscular mycorrhizae for growth promotion and nutrient fortification in soybean under drought conditions. Front Plant Sci. 2022;13:947547. doi: 10.3389/fpls.2022.947547. 35937362 PMC9355629

[9] Ramzan T, Shahbaz M, Maqsood MF, Zulfiqar U, Saman RU, Lili N, Irshad M, Maqsood S, Haider A, Shahzad B, et al. Phenylalanine supply alleviates drought stress in mustard (Brassica campestris) by modulating plant growth, photosynthesis, and antioxidant defense system. Plant Physiol Biochem. 2023;201:107828. doi: 10.1016/j.plaphy.2023.107828. 37329687

[10] Wu L, Zheng Y, Liu S, Jia X, Lv H. Response of ammodendron bifolium seedlings inoculated with AMF to drought stress. Atmos. 2023;14:989.

[11] Gilani M, Danish S, Ahmed N, Rahi AA, Akrem A, Younis U, Irshad I, Iqbal RK. Mitigation of drought stress in spinach using individual and combined applications of salicylic acid and potassium. Pak J Bot. 2020;52:1505–1513. doi: 10.30848/PJB2020-5(18).

[12] Zafar-ul-Hye M, Danish S, Abbas M, Ahmad M, Munir T. ACC-deaminase producing PGPR agrobacterium fabrum and bacillus amyloliquefaciens along with biochar improve wheat productivity under drought stress. Agronomy. 2019;9:343.

[13] Alotaibi MO, Ikram M, Alotaibi NM, Hussain GS, Ghoneim AM, Younis U, Naz N, Danish S. Examining the role of AMF-Biochar in the regulation of spinach growth attributes, nutrients concentrations, and antioxidant enzymes in mitigating drought stress. Plant Stress. 2023;10:100205. doi: 10.1016/j.stress.2023.100205.

[14] Rao MJ, Zheng B. The role of polyphenols in abiotic stress tolerance and their antioxidant properties to scavenge reactive oxygen species and free radicals. Antioxidants. 2025;14:74. doi: 10.3390/antiox14010074. 39857408 PMC11761259

[15] Xiang K, Li Y, Horton R, Feng H. Similarity and difference of potential evapotranspiration and reference crop evapotranspiration-a review. Agric Water Manage. 2020;232:106043.

[16] Patel J, Mishra A. Plant aquaporins alleviate drought tolerance in plants by modulating cellular biochemistry, root-architecture and photosynthesis. Physiol Plant. 2021;172:1030–1044. doi: 10.1111/ppl.13324. 33421148

[17] Das KR, Jahan M, Barman LR, Burman P. Modeling and forecasting of spinach production in Bangladesh. Nep J Stat. 2023;7:1–18. doi: 10.3126/njs.v7i1.61053.

[18] Ma X, Yu LA, Fatima M, Wadlington WH, Hulse-Kemp AM, Zhang X, Zhang S, Xu X, Wang J, Huang H, et al. The spinach YY genome reveals sex chromosome evolution, domestication, and introgression history of the species. Genome Biol. 2022;23:75. doi: 10.1186/s13059-022-02633-x. 35255946 PMC8902716

[19] Hussain N, Ali H, Mustafa G, Sarwar Khan M, Ali B, Ameer S, Zamir S, Iqbal R, Ali B, Khan MN, et al. In vitro plant regeneration from petioles of spinach (*Spinacia oleracea* L.). ACS Agric Sci Technol. 2023;4:57–62. doi: 10.1021/acsagscitech.3c00283.

[20] Ramaiyan B, Kour J, Nayik GA, Anand N, Alam MS. Spinach (*Spinacia oleracea* L.). In GA Nayik, A Gull (Eds.), Antioxidants in Vegetables and Nuts - Properties and Health Benefits. 2020. pp. 159–173 Singapore: Springer. doi: 10.1007/978-981-15-7470-2_8.

[21] Elvira-Torales LI, Periago MJ, Gonzalez-Barrio R, Hidalgo N, Navarro-Gonzalez I, Garcia-Alonso C. Spinach consumption ameliorates the gut microbiota and dislipaemia in rats with diet-induced non-alcoholic fatty liver disease (NAFLD). Food Funct. 2019;10:2148–2160. doi: 10.1039/C8FO01630E. 30938723

[22] Olagoke O. Phytochemical analysis and antibacterial activities of spinach leaf. Am J Phytomed Clin Therapeutics. 2018;6:8.

[23] Shi A, Qin J, Mou B, Correll J, Weng Y, Brenner D, Feng C, Motes D, Yang W, Dong L, et al. Genetic diversity and population structure analysis of spinach by single-nucleotide polymorphisms identified through genotyping-by-sequencing. Publ Libr Sci One. 2017;12:188745. doi: 10.1371/journal.pone.0188745. PMC5708663 29190770

[24] Jabeen M, Akram NA, Ashraf M, Aziz A. Assessment of biochemical changes in spinach (*Spinacia oleracea* L.) subjected to varying water regimes. Sains Malaysiana. 2019;48:533–541. doi: 10.17576/jsm-2019-4803-05.

[25] Aslam Z, Ahmed B, Hafeez S. Optimizing spinach growth through foliar application of plant growth regulators. Indus J Soc Sci. 2023;1:39–42. doi: 10.59075/ijss.v1i02.83.

[26] Fareed S, Haider A, Ramzan T, Ahmad M, Younis A, Zulfiqar U, Rehman HU, Waraich EA, Abbas A, Chaudhary T, et al. Investigating the growth promotion potential of biochar on pea (Pisum sativum) plants under saline conditions. Sci Rep. 2024;14(1):10870. doi: 10.1038/s41598-024-59891-x. 38740776 PMC11091058

[27] Shohat H, Eliaz NI, Weiss D. Gibberellin in tomato: metabolism, signaling and role in drought responses. Mol Hortic. 2021;1:1–12. doi: 10.1186/s43897-021-00019-4. 37789477 PMC10515025

[28] Ali Muhammad, Zulfiqar Faisal, Alghanem Suliman Mohammed Suliman, Fayad Eman, Binjawhar Dalal Nasser, Alqurashi Mohammed, El‐Shaboury Gamal Awad, El‐kott Attalla F. Proline Induced Modulation in Gas Exchange and Antioxidant Defense of Aniseed (*Pimpinella anisum* L.) Seedlings Exposed to Cadmium Stress. New Zealand Journal of Crop and Horticultural Science. 2026;54 10.1002/nzc2.70057.

[29] Betts NS, Dockter C, Berkowitz O, Collins HM, Hooi M, Lu Q, Whelan J. Transcriptional and biochemical analyses of gibberellin expression and content in germinated barley grain. J Exp Bot. 2020;71:1870–1884. doi: 10.1093/jxb/erz546. 31819970 PMC7242073

[30] Khan N, Bano A, Zandi P. Effects of exogenously applied plant growth regulators in combination with PGPR on the physiology and root growth of chickpea (*Cicer arietinum*) and their role in drought tolerance. J Plant Interact. 2018;13:239–247. doi: 10.1080/17429145.2018.1471527.

[31] Patel A, Tiwari S, Prasad SM. Modulation of salt stress in paddy field Cyanobacteria with exogenous application of gibberellic acid: growth behavior and antioxidative status. Physiol Mol Biol Plants. 2023;29:51–68. doi: 10.1007/s12298-022-01266-5. 36733835 PMC9886751

[32] Hui Q, Wang M, Wang P, Ma Y, Gu Z, Yang R. Gibberellic acid promoting phytic acid degradation in germinating soybean under calcium lactate treatment. J Sci Food Agric. 2018;98:644–651. doi: 10.1002/jsfa.8509. 28664974

[33] Cheng W, Yin S, Tu Y, Mei H, Wang Y, Yang Y. SlCAND1, encoding cullinassociated Nedd8-dissociated protein 1, regulates plant height, flowering time, seed germination and root architecture in tomato. Plant Mol Biol. 2020;102:537–551. doi: 10.1007/s11103-020-00963-7. 31916084

[34] Kaya AS, Aydinsakir K, Karaguzel UO. Assessment of GA3 and BA application on gerbera cultivation in soilless culture. Int J Agric Environ Food Sci. 2019;3:41–45. doi: 10.31015/jaefs.2019.1.9.

[35] Arnon DI. Copper enzyme in isolated chloroplast. Polyphenol oxidase in *Beta vulgaris*. Plant Physiol. 1949;24:1–15. doi: 10.1104/pp.24.1.1. 16654194 PMC437905

[36] Velikova V, Yordanov I, Edreva A. Oxidative stress and some antioxidant systems in acid rain-treated bean plants: protective roles of exogenous polyamines. Plant Sci. 2000;151:59–66.

[37] Heath RL, Packer L. Photo peroxidation in isolated chloroplast kinetics and stoichiometry of fatty acid peroxidation. Arch Biochem Biophys. 1968;125:189–198. doi: 10.1016/0003-9861(68)90654-1. 5655425

[38] Spitz DR, Oberly LW. Measurement of MnSOD and CuZnSOD activity in mammalian tissue homogenates. Curr Protoc Toxicol. 2001;8:751–758. doi: 10.1002/0471140856.tx0705s08. 20954153

[39] Chance B, Maehly A. Assay of catalase and peroxidase. Methods Enzymol. 1955;2:764–817.

[40] Mukherjee SP, Choudhari MA. Implications for water stress-induced changes in the level of endogenous ascorbic acid and hydrogen peroxide in *Vigna seedlings*. Physiol Plant. 1983;58:116–170. doi: 10.1111/j.1399-3054.1983.tb04162.x.

[41] Strack D, Wray V. Anthocyanins. In JB Harborne (Ed.), Methods in plant biology, Plant Phenolics. 1989. Vol. 1, 325–356. Academic Press. doi: 10.1016/B978-0-12-461011-8.50015-9.

[42] Kim JH, Jeon SM, Park YA, Choi MS, Moon KD. Effects of safflower seed (*C**arthamus tinctorius* L.) powder on lipid metabolism in high fat and high cholesterol-fed rats. J Korean Soc Food Sci Nutr. 1999;28:625–631.

[43] Julkenen-Titto R. Phenolic constituents in the leaves of Northern willows: methods for the analysis of certain phenolics. J Agric Food Chem. 1985;33:213–217. doi: 10.1021/jf00062a013.

[44] Bradford MM. A rapid and sensitive method for the quantitation of microgram quantities of protein utilizing the principle of protein-dye binding. Anal Biochem. 1976;72:248–254. doi: 10.1016/0003-2697(76)90527-3. 942051

[45] Yoshida S, Forno D, Cock J, Gomez K. Determination of sugars and starch in plant tissue. In Laboratory manual for physiological studies of rice. 1976. Vol. 3, 46–49. International Rice Research Institute.

[46] Lavezo A, Cargnelutti A, Bem CMD, Burin C, Kleinpaul JA, Pezzini RV. Plot size and number of replications to evaluate the grain yield in oat cultivars. Bragantia. 2017;76:512–520.

[47] Kausar A, Zahra N, Tahir H, Hafeez MB, Abbas W, Raza A. Modulation of growth and biochemical responses in spinach (*Spinacia oleracea* L.) through foliar application of some amino acids under drought conditions. S Afr J Bot. 2023;158:243–253. doi: 10.1016/j.sajb.2023.05.018.

[48] Tajdari HR, Soleymani A, Montajabi N, Naderi Darbaghshahi MR, Javanmard HR. The effect of foliar application of plant growth regulators on functional and qualitative characteristics of wheat (*Triticum aestivum* L.) under salinity and drought stress conditions. Appl Water Sci. 2024;14:1–15. doi: 10.1007/s13201-024-02203-5.

[49] Kausar A, Zahra N, Zahra H, Hafeez MB, Zafer S, Shahzadi A, Raza A, Djalovic I, Prasad PV. Alleviation of drought stress through foliar application of thiamine in two varieties of pea (*Pisum sativum* L.). Plant Signal Behav. 2023;18:2186045. doi: 10.1080/15592324.2023.2186045. 37016728 PMC10012936

[50] Haque MN, Pramanik SK, Islam MHM, Sikder S. Foliar application of potassium and gibberellic acid (GA3) to alleviate drought stress in wheat. J Sci Technol. 2022;20:1994–0386. doi: 10.59125/JST.20201.

[51] Elahi NN, Raza S, Rizwan MS, Albalawi BFA, Ishaq MZ, Ahmed HM, Mehmood S, Imtiaz M, Farooq U, Rashid M, et al. Foliar application of gibberellin alleviates adverse impacts of drought stress and improves growth, physiological and biochemical attributes of canola (*Brassica napus* L.). Sustainability. 2022;15:78. doi: 10.3390/su15010078.

[52] Xu C, Leskovar DI. Effects of A. Nodosum seaweed extracts on spinach growth, physiology and nutrition value under drought stress. Sci Hortic (Amsterdam). 2015;183:39–47.

[53] Tefera A, Kebede M, Tadesse K, Getahun T. Morphological, physiological and biochemical characterization of drought-tolerant wheat (*Triticum* spp.) varieties. Int J Agron. 2021;2021:8811749.

[54] Farooq M, Wahid A, Kobayashi NSMA, Fujita DBSMA, Basra SM. Plant drought stress: effects, mechanisms and management. Sustain Agric. 2009;29:153–188. doi: 10.1007/978-90-481-2666-8_12.

[55] Bandian L, Saeb H, Abedy B. Effect of bentonite on growth indices and physiological traits of spinach (*Spinacia oleracea* L.) under drought stress. J Product Dev. 2016;2:1–6.

[56] Das D, Bhadra AK, Moniruzzaman M. Foliar spray of gibberellic acid influences morphological attributes and foliage yield of coriander (*Coriandrum sativum* L.). Res Agric Livest Fisheries. 2018;5:1–9. doi: 10.3329/ralf.v5i1.36546.

[57] Iftikhar A, Rizwan M, Adrees M, Ali S, ur Rehman MZ, Qayyum MF, Hussain A. Effect of gibberellic acid on growth, biomass, and antioxidant defense system of wheat (*Triticum aestivum* L.) under cerium oxide nanoparticle stress. Environ Sci Pollut Res. 2020;27:33809–33820. doi: 10.1007/s11356-020-09661-9. 32535824

[58] Ramesh S, Sudhakar P, Elankavi S, Suseendran K, Jawahar S. Effect of gibberellic acid (GA3) on growth and yield of rice (*Oryza sativa* L.). Plant Archives. 2019;19:1369–1372.

[59] Dalal VK. Modulation of photosynthesis and other proteins during water-stress. Mol Biol Rep. 2021;48:3681–3693. doi: 10.1007/s11033-021-06329-6. 33856605

[60] Seymen M. Comparative analysis of the relationship between morphological, physiological and biochemical properties in spinach (*Spinacia oleracea* L.) under deficit irrigation conditions. Turk J Agric For. 2021;45:55–67.

[61] Usman M, Raheem Z, Ahsan T, Iqbal A, Sarfaraz ZN, Haq Z. Morphological, physiological and biochemical attributes as indicators for drought tolerance in rice (*Oryza sativa* L.). Eur J Biol Sci. 2013;5:23–28.

[62] Banks JM. Chlorophyll fluorescence as a tool to identify drought stress in Acer genotypes. Environ Exp Bot. 2018;155:118–127. doi: 10.1016/j.envexpbot.2018.06.022.

[63] Miri M, Ghooshchi F, Tohidi-Moghadam HR, Larijani HR, Kasraie P. Ameliorative effects of foliar spray of glycine betaine and gibberellic acid on cowpea (*Vigna unguiculata* L. Walp.) yield affected by drought stress. Arabian J Geosci. 2021;14:830. doi: 10.1007/s12517-021-07228-7.

[64] Divashuk MG, Kroupin PY, Shirnin SY, Vukovic M, Kroupina AY, Karlov GI. Effect of gibberellin responsive reduced height allele Rht13 on agronomic traits in spring bread wheat in field experiment in non-black soil zone. Agronomy. 2020;10:927. doi: 10.3390/agronomy10070927.

[65] Hasanuzzaman M, Bhuyan MB, Zulfiqar F, Raza A, Mohsin SM, Mahmud JA, Fujita M, Fotopoulos V. Reactive oxygen species and antioxidant defense in plants under abiotic stress: revisiting the crucial role of a universal defense regulator. Antioxidants. 2020;9:681. doi: 10.3390/antiox9080681. 32751256 PMC7465626

[66] Pandey J, Devadasu E, Saini D, Dhokne K, Marriboina S, Raghavendra AS, Subramanyam R. Reversible changes in structure and function of photosynthetic apparatus of pea (*Pisum sativum*) leaves under drought stress. Plant J. 2023;113:60–74. doi: 10.1111/tpj.16034. 36377283

[67] Liu J, Wang J, Lee S, Wen R. Copper-caused oxidative stress triggers the activation of antioxidant enzymes via ZmMPK3 in maize leaves. Publ Libr Sci One. 2018;13:0203612. doi: 10.1371/journal.pone.0203612. PMC6141078 30222757

[68] Saleem MH, Fahad S, Adnan M, Ali M, Rana MS, Kamran M, Ali Q, Hashem IA, Bhantana P, Ali M, et al. Foliar application of gibberellic acid endorsed phytoextraction of copper and alleviates oxidative stress in jute (*Corchorus capsularis* L.) plant grown in highly copper-contaminated soil of China. Environ Sci Pollut Res. 2020;27:37121–37133. doi: 10.1007/s11356-020-09764-3. 32583108

[69] Dien DC, Mochizuki T, Yamakawa T. Effect of various drought stresses and subsequent recovery on proline, total soluble sugar and starch metabolisms in rice (*Oryza sativa* L.) varieties. Plant Prod Sci. 2019;22:530–545. doi: 10.1080/1343943X.2019.1647787.

[70] Bukhari SABH, Lalarukh I, Amjad SF, Mansoora N, Naz M, Naeem M, Bukhari SA, Shahbaz M, Ali SA, Marfo TD, et al. Drought stress alleviation by potassium-nitrate-containing chitosan/montmorillonite microparticles confers changes in *spinacia oleracea* L. Sustainability. 2021;13:9903. doi: 10.3390/su13179903.

[71] Zhang X, Ma M, Wu C, Huang S, Danish S. Mitigation of heat stress in wheat (Triticum aestivum L.) via regulation of physiological attributes using sodium nitroprusside and gibberellic acid. BMC Plant Biol. 2023;23:302. 37280509 10.1186/s12870-023-04321-9 PMC10242961

[72] Rady MM, Boriek SH, Abd El-Mageed TA, Seif El-Yazal MA, Ali EF, Hassan FA, Abdelkhalik A. Exogenous gibberellic acid or dilute bee honey boosts drought stress tolerance in *Vicia faba* by rebalancing osmoprotectants, antioxidants, nutrients and phytohormones. Plants. 2021;10:748. doi: 10.3390/plants10040748. 33920494 PMC8068922

[73] Xu W, Cui K, Xu A, Nie L, Huang J, Peng S. Drought stress condition increases root to shoot ratio via alteration of carbohydrate partitioning and enzymatic activity in rice seedlings. Acta Physiol Plant. 2015;37:1–11.

[74] Shahzad K, Hussain S, Arfan M, Hussain S, Waraich EA, Zamir S, Saddique M, Rauf A, Kamal KY, Hano C, et al. Exogenously applied gibberellic acid enhances growth and salinity stress tolerance of maize through modulating the morpho-physiological, biochemical and molecular attributes. Biomolecules. 2021;11:1005. doi: 10.3390/biom11071005. 34356629 PMC8301807

[75] Kuromori T, Seo M, Shinozaki K. ABA transport and plant water stress responses. Trends Plant Sci. 2018;23:513–522. doi: 10.1016/j.tplants.2018.04.001. 29731225

[76] Bhuiyan TF, Ahamed KU, Nahar K, Al-Mahmud J, Bhuyan MB, Anee TI, Hasanuzzaman M. Mitigation of PEG-induced drought stress in rapeseed (*B**rassica rapa* L.) by exogenous application of osmolytes. Biocatal Agric Biotechnol. 2019;20:101197. doi: 10.1016/j.bcab.2019.101197.

[77] Guo X, Wu Q, Zhu G, Hussien Ibrahim ME, Zhou G. Gibberellin increased yield of sesbania pea grown under saline soils by improving antioxidant enzyme activities and photosynthesis. Agronomy. 2022;12:1855. doi: 10.3390/agronomy12081855.

[78] Naikoo MI, Dar MI, Raghib F, Jaleel H, Ahmad B, Raina A, Khan FA, Naushin F. Role and regulation of plants phenolics in abiotic stress tolerance: an overview. Plant Signal Mol. 2019;35:157–168. doi: 10.1016/B978-0-12-816451-8.00009-5.

[79] Nahar K, Hasanuzzaman M, Fujita M. Roles of osmolytes in plant adaptation to drought and salinity. In Osmolytes and plants acclimation to changing environment. Emerging Omics Technologies. 2016 3, pp. 37–68.

[80] Sakr MT, Ibrahim HM, ElAwady AE, AboEL-Makarm AA. Growth, yield and biochemical constituents as well as post-harvest quality of water-stressed broccoli (*Brassica oleracea* L. var. italica) as affected by certain biomodulators. Sci Hortic (Amsterdam). 2021;275:109605. doi: 10.1016/j.scienta.2020.109605.

[81] Qamer Z, Chaudhary MT, Du X, Hinze L, Azhar MT. Review of oxidative stress and antioxidative defense mechanisms in *Gossypium hirsutum* L. In response to extreme abiotic conditions. J Cotton Res. 2021;4:9. doi: 10.1186/s42397-021-00086-4.

[82] Jan R, Aaqil Khan M, Asaf S, Lubna JR, Park, Lee IJ, Kim KM. Flavonone 3-hydroxylase relieves bacterial leaf blight stress in rice via over-accumulation of antioxidant flavonoids and induction of defense genes and hormones. Int J Mol Sci. 2021;22:6152. doi: 10.3390/ijms22116152. 34200345 PMC8201380

[83] Tripathi JM, Khan BR, Gaur R, Yadav D, Verma KK, Gupta R. Gibberellic acid improves photosynthetic electron transport and stomatal function in crops that are adversely affected by salinity exposure. Plants. 2025;14(21):3388. doi: 10.3390/plants14213388. 41225938 PMC12608142

[84] Hm M, Doddagoudar S, Gowda B, Nm S. Influence of seed priming and foliar spray on seed germination, seedling growth, total carbohydrate and protein content of resultant rice (Oryza Sativa L.) seeds under salinity. Seed Res. 2024;48(1):49–54.

[85] Anwar T, Qureshi H, El-Beltagi HS, Shokirov A, Khudoyberdiyeva N, Nurniyazov A, Mamarakhimov O, Rebouh NY, Alomran MM, Alsudays IM, et al. Biochar and GA3-mediated enhancement of wheat performance under combined salinity and drought: physiological and biochemical insights for resilient agriculture. Biodegradation. 2025;37. doi: 10.1007/s10532-025-10225-2. 41331392

